# Financial Toxicity in Women with Endometriosis: Psychometric Validation of the Polish COST-FACIT with Analysis of Demographic and Clinical Factors

**DOI:** 10.3390/healthcare14111449

**Published:** 2026-05-24

**Authors:** Katarzyna Pietrzak, Anna Weronika Szablewska, Arkadiusz Prajzner, Aleksandra Gaworska-Krzemińska, Bartosz Pryba

**Affiliations:** 1 Independent Monoprofile Medical Simulation Laboratory, Institute of Nursing and Midwifery, Medical University of Gdansk, Sklodowskiej-Curie 3A, 80-210 Gdansk, Poland; bartosz.pryba@gumed.edu.pl; 2 Department of Obstetric and Gynaecological Nursing, Institute of Nursing and Midwifery, Medical University of Gdansk, Sklodowskiej-Curie 3A, 80-210 Gdansk, Poland; anna.szablewska@gumed.edu.pl; 3 Institute of Psychology, Faculty of Pedagogy and Psychology, University of the National Education Commission, 30-084 Krakow, Poland; arkadiusz.prajzner@uken.krakow.pl; 4 Division on Nursing Management, Institute of Nursing and Midwifery, Medical University of Gdansk, Sklodowskiej-Curie 3A, 80-210 Gdansk, Poland; aleksandra.gaworska-krzeminska@gumed.edu.pl

**Keywords:** financial toxicity, COST-FACIT, questionnaire validation, psychometric properties, endometriosis

## Abstract

Background: Endometriosis is a chronic condition associated with substantial healthcare costs, diagnostic delays and long-term impairment in quality of life. Despite the recognized economic burden, patient-reported financial distress remains insufficiently studied. The aim of this study was to adapt and validate the Polish version of the Comprehensive Score for Financial Toxicity (COST-FACIT) for use in women with endometriosis, as well as to examine demographic and clinical factors associated with financial toxicity. Methods: A cross-sectional study was conducted among Polish women with endometriosis using an online survey. The COST-FACIT was adapted following standard forward–backward translation procedures, with FACIT approval. Psychometric evaluation included internal consistency, construct validity, convergent validity with the Financial Well-Being Scale, and test–retest reliability. Exploratory and confirmatory factor analyses were performed, and multivariable models were used to identify factors associated with financial toxicity. Results: The adapted scale demonstrated good psychometric properties, with excellent internal consistency (Cronbach’s α = 0.92; McDonald’s ω = 0.92) and strong test–retest reliability (r = 0.87). Exploratory factor analysis supported a two-factor structure of the instrument. COST-FACIT scores were strongly correlated with financial well-being (r = 0.78). Higher education, stable employment and higher income were associated with better financial well-being, whereas longer symptom duration, greater distance to care and higher healthcare expenditures were associated with worse scores. Conclusions: The Polish COST-FACIT demonstrated good psychometric properties and may serve as a useful instrument for assessing financial toxicity in women with endometriosis. The results highlight the financial burden of the disease and support the use of patient-reported measures to identify individuals at risk of financial distress and reduced quality of life. This tool may facilitate clinical research and improve patient-centered care.

## 1. Introduction

Endometriosis is a chronic, progressive and often debilitating condition affecting a substantial proportion of women of reproductive age worldwide. Globally, approximately 10% of women of reproductive age are estimated to suffer from endometriosis, corresponding to nearly 190 million individuals [1]. In Poland, reliable epidemiological estimates remain limited due to the lack of a national endometriosis registry; however, available data suggest a growing number of diagnosed cases [2,3]. In addition, expert estimates from the Polish Medical Research Agency [Agencja Badań Medycznych, ABM] suggest that endometriosis may affect up to approximately three million women in Poland, underscoring the potentially large but insufficiently quantified scale of the problem [2]. The disease is commonly associated with persistent pelvic pain and infertility, as well as a considerable reduction in daily functioning and quality of life, collectively imposing a significant personal and societal burden. The economic impact of endometriosis arises not only from direct medical expenses—including frequent consultations, pharmacological treatment, diagnostic procedures and surgical interventions—but also from indirect costs such as work absenteeism, reduced productivity and long-term disability [4]. Although the economic burden of endometriosis has been widely documented in cost-of-illness studies, this literature primarily reflects aggregate expenditure rather than the individual patient experience, leaving the concept of subjective financial strain insufficiently explored in this population [5].

Diagnosing endometriosis remains challenging. Laparoscopy with histopathological confirmation is still considered the diagnostic gold standard, although high-quality imaging is increasingly used in specialized centers. Substantial diagnostic delays have been regularly reported in the literature. Studies consistently report diagnostic delays that often exceed 6–10 years [6]. During this prolonged period, many patients consult multiple physicians, receive alternative diagnoses and undergo repeated investigations, all of which further amplify the physical, psychological and economic burden associated with the disease [7,8,9].

In oncology, the concept of financial toxicity—defined as the financial distress and hardship caused by illness and its treatment—has been thoroughly explored. The Comprehensive Score for Financial Toxicity (COST-FACIT) has emerged as the most widely used and best-validated instrument for its assessment. The original validation study demonstrated good internal consistency, test–retest reliability and construct validity in patients with cancer, and subsequent cross-cultural adaptations across multiple languages and settings have consistently confirmed its psychometric robustness [10,11,12].

To date, the COST-FACIT has not been applied in the case of women with endometriosis, and the extent of financial toxicity in this population remains insufficiently explored. Although the COST-FACIT was originally designed for oncology, recent evidence demonstrates its successful adaptation and psychometric validation in other chronic conditions—notably diabetes, where it allowed reliable capture of financial distress and stratification of patients into meaningful toxicity classes [11,12]. This evidence supports the applicability of the COST-FACIT in endometriosis, highlighting the need for its validation to enable a structured assessment of financial toxicity in this population. A validated instrument could facilitate the identification of patient subgroups at increased risk of financial distress, inform reimbursement and resource allocation policies while also supporting future research on economic burden, inequality and patient support interventions [1,4,13,14].

The primary aim of this study was to evaluate the psychometric properties of the Polish version of the COST-FACIT. A secondary, exploratory aim was to examine associations between financial toxicity and selected demographic and clinical variables.

## 2. Material and Methods

### 2.1. Study Design and Participants

The factor structure and psychometric properties of the Polish version of the COST-FACIT were evaluated in this cross-sectional observational study among a cohort of women diagnosed with endometriosis. The study was conducted in accordance with the 1964 Declaration of Helsinki, and its later amendments and approved by the Bioethics Committee of the Medical University of Gdańsk (KB/517-460/2025). During the fourth quarter of 2025, a total of 983 questionnaires were administered, of which 507 were completed and eligible for further assessment. Following eligibility verification, 504 women were included in the final evaluation. Participants were recruited through online patient communities, social media support groups, endometriosis advocacy networks, and a collaborating Women’s Health Center where the study invitation was disseminated among patients receiving care at the clinic. Given the online recruitment strategy, the sample likely included participants from different regions of Poland; however, detailed geographical distribution was not formally established. The study population consisted of individuals with a confirmed or strongly suspected diagnosis of endometriosis. In accordance with current international guidelines (e.g., ESHRE 2022) [15], the study focused on reproductive-age patients assigned female sex at birth, as this group represents the vast majority of those affected in the clinical setting. Although endometriosis can occur in transgender, non-binary or gender-diverse individuals, such cases are rare in the Polish healthcare context and were therefore not analyzed as a separate subgroup. Importantly, no participant was excluded based on gender identity, ethnicity or cultural background; eligibility was determined solely by clinical criteria and the ability to provide informed consent. Participation was self-initiated, and respondents were not contacted directly by the researchers. Before accessing the questionnaire, all potential participants were provided with information about the study aims, eligibility criteria and data confidentiality and were required to provide electronic informed consent. The demographic and clinical characteristics of the study sample are presented in Table 1.

The survey was available to any eligible individual, and no financial incentives were offered. This online, community-based strategy was chosen to reach a broad population of individuals affected by endometriosis, including those not currently engaged in specialist care. Potential sources of bias associated with this recruitment approach are addressed in Section Bias and Section Limitations.

Participants were eligible if they met all the following criteria:

- age ≥ 18 years;
- ability to provide electronic informed consent and complete the online questionnaire;
- self-reported diagnosis of endometriosis previously established by a healthcare professional in accordance with current international clinical recommendations (e.g., ESHRE 2022, ACOG, RCOG), defined as:
  (a) definite diagnosis—confirmed by a healthcare professional based on laparoscopy with or without histopathological verification, and
  (b) clinically suspected diagnosis—communicated by a healthcare professional, based on characteristic findings on pelvic imaging (ultrasound and/or MRI), and/or typical symptoms consistent with endometriosis (e.g., dysmenorrhea, chronic pelvic pain, dyspareunia, cyclic urinary or bowel symptoms, infertility), with no alternative explanation identified during prior clinical assessment (this definition reflects current international guideline statements indicating that laparoscopy is not re-quired for routine diagnosis when clinical and imaging findings are consistent with endometriosis).

All eligibility criteria and the operational definition of endometriosis diagnosis were explicitly described in the study invitation and introductory survey materials to ensure that respondents understood the basis for participation. Endometriosis was defined as either a clinically confirmed diagnosis (based on imaging and/or surgical findings) or a strongly suspected diagnosis established by a physician according to current international guidelines (e.g., ESHRE 2022). In cases where questionnaire responses raised uncertainty regarding eligibility, the information provided was reviewed by healthcare professionals involved in the study. Ambiguous cases were evaluated against international guideline criteria to determine whether inclusion was appropriate.

Participants were excluded if any of the following criteria were met:

- no prior medical evaluation related to endometriosis, i.e., respondents who reported self-diagnosis only without any assessment or communication from a healthcare professional;
- symptoms alone, without imaging or clinical suspicion, meaning that endometriosis had not been previously considered, suggested or investigated by a clinician, and no imaging consistent with endometriosis had ever been performed;
- previously established alternative diagnosis that could plausibly explain pelvic pain or related symptoms (e.g., malignant disease, acute pelvic infection, inflammatory bowel disease, interstitial cystitis, and urological disorders), as self-reported;
- current pregnancy or breastfeeding at the time of survey completion, due to the potential impact on symptom profile and clinical interpretation;
- inability to provide electronic informed consent or failure to complete essential sections of the questionnaire, preventing reliable analysis;
- duplicate submissions identified through internal data-quality checks, with only the first complete response retained;
- cases in which the available questionnaire information was insufficient to determine eligibility and where subsequent review by healthcare professionals (based on international guideline criteria, e.g., ESHRE 2022) indicated that inclusion could not be justified.

**Figure 1:**
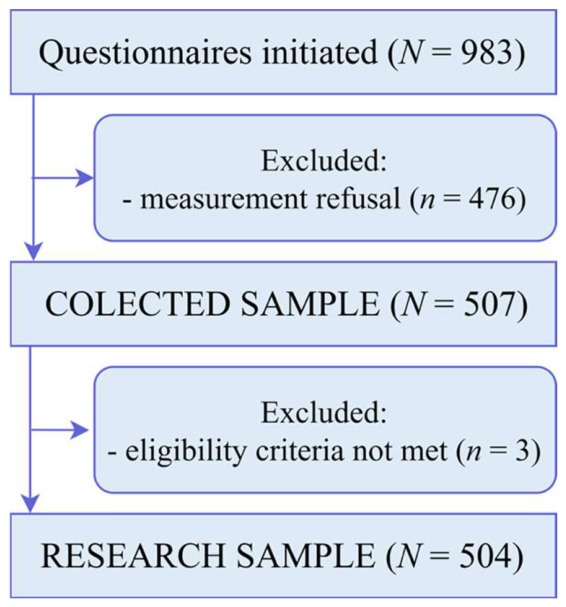
Flow of participants throughout the study.

The flow of participants throughout the study is illustrated in Figure 1.

#### Bias

Several potential sources of bias were considered in the design and conduct of this study. Selection bias may have arisen due to the online, voluntary nature of recruitment, as individuals who were more engaged in patient support networks or experiencing more severe symptoms may have been more likely to participate. Diagnostic misclassification bias could be possible because endometriosis status was self-reported rather than clinically verified within the study. To minimize this risk, the inclusion criteria reflected international guideline definitions (e.g., ESHRE 2022) [15], and responses that raised uncertainty were reviewed by healthcare professionals. Participants reporting self-diagnosis without any prior medical evaluation were excluded.

Information bias, particularly related to recall and reporting, may have occurred due to the self-reported data on symptoms and clinical history. To limit this, standardized wording, closed-ended items and clear definitions were used in the questionnaire, and participants completed the survey independently, without researcher influence.

Non-response and incomplete data bias could have occurred if individuals had discontinued the survey or omitted key items. Only complete responses meeting eligibility criteria were included, and duplicate submissions were removed through internal data-quality checks. Confounding cannot be fully excluded, as variables such as comorbidities, treatment history or socioeconomic factors may influence symptom perception and reporting. Relevant demographic and clinical variables were collected to allow descriptive assessment; however, causal inference was not the aim of the study.

Despite these measures, the findings should be interpreted with the awareness that the sample represents an online, self-selected population rather than a clinically enrolled cohort.

### 2.2. Data Collection

Data were collected using an online survey. The survey was distributed through a unique access link, and responses were submitted using an encrypted HTTPS connection to ensure the protection of participant data. No identifying personal information was collected unless participants voluntarily provided an e-mail address for the purpose of the retest procedure. In such cases, e-mail data were stored separately from survey responses to preserve confidentiality. The survey was anonymous for participants not taking part in the re-test phase. Access was not restricted by tokens, allowing a broad and inclusive response range. All data were stored on university servers and processed in compliance with GDPR regulations and the study’s bioethical approval conditions.

1. Sociodemographic and socioeconomic profile

Data were collected on sociodemographic and socioeconomic characteristics, including age, place of residence, education level, employment status, household income and selected indicators of social support.

2. Diagnostic history of endometriosis

Information was collected on the diagnostic history of endometriosis, including timing of diagnosis, diagnostic delay and diagnostic procedures, to allow characterization of the study population.

3. Treatment history and cost of management

Data on treatment history and disease-related costs were collected, including medical and non-medical management strategies and patient-reported out-of-pocket expenditures, to describe the clinical and economic context of the study population. All cost-related data were self-reported and referred to cumulative personal expenses. Participants were asked to estimate total spending on endometriosis-related care, including pharmacological treatment, medical consultations, surgical procedures, infertility treatment and diagnostic services. These measures reflect direct medical costs. Indirect costs were not assessed.

### 2.3. Standardized Psychometric Instruments

#### 2.3.1. Financial Well-Being Scale (FWBS)

Financial well-being was assessed using the validated Financial Well-Being Scale, which measures subjective financial security, perceived control over expenses, savings resilience and emotional responses to financial strain. Higher scores indicate better financial well-being and lower subjective financial distress. The FWBS has demonstrated strong internal consistency in previous psychometric studies, with Cronbach’s α reported at 0.89 in a Portuguese validation sample [16]. The scale, originally developed by the Consumer Financial Protection Bureau (CFPB), has previously been translated and applied in a Polish online survey [17]. In the present study, the Polish-language version was used and scored according to standard procedures described in the CFPB technical report [18].

#### 2.3.2. COST-FACIT—Financial Toxicity

Financial toxicity was assessed using the Comprehensive Score for Financial Toxicity (COST), originally developed by de Souza et al. [19] within the Functional Assessment of Chronic Illness Therapy (FACIT) system. The instrument captures the multidimensional burden of treatment-related financial stress, including perceived economic strain, concerns about future financial stability, work impairment, emotional burden and perceived control over financial resources. The COST consists of 12 items scored on a five-point Likert scale (0 = not at all to 4 = very much), summed to yield a total score, with reverse scoring applied where appropriate and in accordance with the FACIT manual. The scale is conceptualized as unidimensional and is therefore reported as a total score without subscales. Higher scores indicate lower financial toxicity (greater financial stability), whereas lower scores reflect greater financial strain. In previous validation studies, strong internal consistency of the COST-FACIT has been demonstrated in both oncological and non-oncological populations (Cronbach’s α = 0.86–0.92), and subsequent language adaptations have confirmed good reliability (α = 0.77–0.88) [12,19]. The COST-FACIT served as the primary outcome measure of financial toxicity in this study and was evaluated for internal consistency, test–retest reliability, and convergent validity against the Financial Well-Being Scale. The use of the COST-FACIT in this study was conducted under license from the FACIT Group [19]. The adaptation of the instrument for use in endometriosis, including cross-cultural translation and disease-specific wording adjustments, is described in detail in the ‘Adaptation and validation process’ section.

### 2.4. Adaptation and Validation Process

The adaptation of the COST-FACIT followed international guidelines for cross-cultural validation and consisted of multiple controlled stages designed to ensure semantic, conceptual and cultural equivalence with the original instrument. Two independent native Polish translators produced separate forward translations of the original English COST-FACIT. These versions were subsequently reconciled into a single consensus version based on comparison with the source text and conceptual accuracy. The reconciled Polish version was then back-translated into English by an independent bilingual translator blinded to the original wording. The complete translation package, including forward translations, the reconciled version and the back-translation, was reviewed by the FACIT translation coordinator, who provided formal feedback and required clarifications. All comments were addressed during the language coordination stage to ensure item-level semantic accuracy and conceptual equivalence. Following linguistic approval, the Polish version was formatted according to the official FACIT questionnaire layout and subjected to cognitive debriefing with representatives of the target population to confirm clarity, acceptability and comprehension of all items.

Throughout the adaptation process, no structural or conceptual modifications were introduced beyond linguistic adjustments. The translation received formal approval from the FACIT Group, and the final Polish version was authorized for research use under license. With explicit permission from FACIT, references to “cancer” in selected items were replaced with the broader term “illness” to ensure contextual appropriateness for endometriosis, while preserving conceptual equivalence with the original scale. Additionally, with the approval of the FACIT Group, gender-inclusive feminine forms were used in the Polish version to reflect grammatical norms and gendered language conventions specific to Polish. Psychometric evaluation of the adapted version included assessment of internal consistency as well as construct and convergent validity against the Financial Well-Being Scale [20].

#### Re-Test

To assess the temporal stability of the instrument, a re-test procedure was conducted. Participants were invited to complete the questionnaire a second time. In the re-test phase, a total of 173 questionnaires were initiated, of which 97 were completed. After data verification, duplicate responses from the same participants were identified and excluded, resulting in a final re-test sample of 82 participants included in the test–retest reliability analysis.

Test–retest reliability was assessed based on paired responses obtained from these participants, with an interval of 7–10 days between the first and second administrations of the questionnaire. The test–retest interval was selected in accordance with methodological recommendations, as it is considered sufficiently short to minimize true changes in the measured construct while long enough to reduce recall bias [14,21]. Responses were matched using a participant-provided email address, which was stored separately from survey data. All analyses were conducted on anonymized, matched identifiers.

### 2.5. Statistical Analysis

The results were analyzed using PS IMAGO PRO version 11.0 and Jamovi version 2.6. For the entire analysis, a significance level of α = 0.05 was adopted. In the psychometric evaluation of the COST-FACIT instrument, both exploratory factor analysis (EFA) and confirmatory factor analysis (CFA) were applied. To reduce the risk of model overfitting, the sample was randomly split into two independent subsamples. Exploratory factor analysis was conducted in the first subsample, whereas confirmatory factor analysis of the revised structure was conducted in the second subsample. The naming of the identified factors was based on the conceptual interpretation of the items loading on each factor and their thematic consistency. The two subdimensions were labeled “Subjective Financial Situation” and “Disease-Related Financial Strain”. The validity of the instrument was also confirmed using Spearman’s r_s_ between COST-FACIT scores and data obtained from an external instrument. Reliability assessment was conducted with respect to internal consistency (Cronbach’s alpha and McDonald’s omega) as well as temporal stability (test–retest correlation). Analysis of potential differences in COST-FACIT scores in relation to demographic and clinical variables was performed using the General Linear Model (GLM). These analyses were considered secondary and exploratory in relation to the primary aim of psychometric validation. A generalized linear model (GLM) was used to account for multiple predictors, including both quantitative and categorical variables, and to provide a flexible framework for modeling the outcome variable.

## 3. Results

### 3.1. Construct Validity

**Figure 2:**
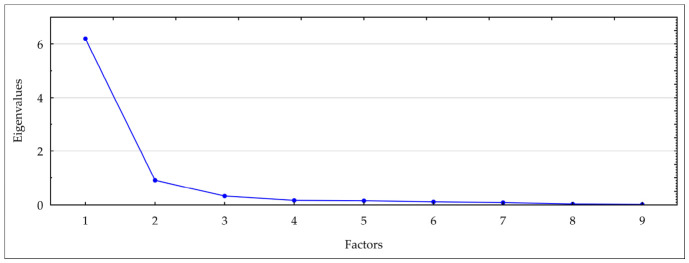
Eigenvalues of COST-FACIT illness [endometriosis] scale.

Construct validity was assessed using factor analysis. Confirmatory factor analysis (CFA) models were estimated using the weighted least squares method. The number of factors and their composition in the two-factor models were determined based on the theoretical framework of the COST-FACIT Diabetes scale (Patel et al., 2022) [12] as well as on the results of exploratory factor analysis (EFA). Exploratory factor analysis was conducted using principal axis factoring with oblimin rotation based on a polychoric correlation matrix. To reduce the risk of model overfitting, exploratory and confirmatory factor analyses were performed in two independent subsamples obtained through random splitting of the study sample. Prior to exploratory factor analysis, sample adequacy was confirmed (n = 257; KMO = 0.91), and the correlation matrix was found suitable for factor analysis (Bartlett’s test: χ^2^(55) = 1498.75, *p* < 0.01). As illustrated in Figure 2, Cattell’s scree criterion supported a two-factor solution.

Factor loadings for the revised COST-FACIT structure are presented in Table 2. Items 1, 6, 7, 8, and 11 loaded primarily on the first factor, labelled Subjective financial situation, whereas items 2, 3, 4, 5, 9, and 10 loaded primarily on the second factor, labelled Disease-related financial strain. The proposed factor structure was subsequently supported by confirmatory factor analysis conducted in an independent subsample (n = 247), with model fit indices presented in Table 3. In addition, a one-factor model was tested due to very strong correlations observed between the factors. A summary of the model fit indices for the tested models is presented in Table 3. The specification of a second-order latent factor representing the overall score within the two-factor models was not feasible due to very strong inter-factor correlations, which led to model identification problems [22].

**Figure 3:**
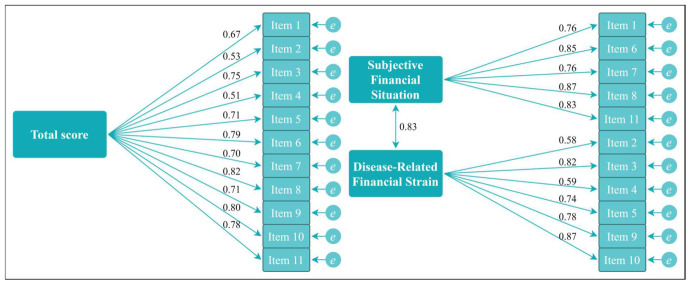
Path diagram of COST-FACIT illness [endometriosis] scale covering standardized coefficients. Note. Two-factor model based on previous Exploratory Factor Analysis.

All tested models demonstrated an acceptable-to-good fit to the data. The one-factor model showed borderline fit, as indicated by an elevated RMSEA value; however, other goodness-of-fit indices, including CFI, TLI and the χ^2^/df ratio, suggested an overall acceptable model fit. The two-factor model based on the theoretical assumptions of the COST-FACIT diabetes structure [13] also exhibited an acceptable fit to the data, as reflected by satisfactory values of CFI, TLI and χ^2^/df. The proposed two-factor COST-FACIT endometriosis model exhibited the best fit to the data among all tested solutions. The factor structures of the tested models are presented in Figure 3.

The two-factor solution presented above further supports the use of the overall COST-FACIT score, given the very strong intercorrelations between the two subscales, labelled subjective financial situation and disease-related financial strain. The acceptable fit of the one-factor model, together with the strong correlations observed between the subscales in the two-factor model, justifies the assessment and interpretation of a total score calculated as the sum of all items. At the same time, the strong fit of the two-factor model (see Table 3 and Figure 3) suggests that the scale may also be used in future research to explore financial burden in more specific domains, namely subjective financial situation and disease-related financial strain. Convergent validity was assessed by examining the associations between COST-FACIT scores and an external measure; the results are summarized in Table 4.

The overall COST-FACIT score as well as the subjective financial situation subscale showed positive and very strong correlations with the external measure of financial well-being. The financial burden subscale correlated with the external instrument less strongly; however, the association remained positive and strong. The results of the factor analysis, including the observed correlations with the external measure, provide preliminary evidence of convergent validity regarding the Polish version of the COST-FACIT illness scale.

### 3.2. Reliability

Internal consistency of the instrument was assessed using Cronbach’s alpha and McDonald’s omega coefficients. The analysis showed that the overall COST-FACIT endometriosis score demonstrated high reliability (Cronbach’s α = 0.92; McDonald’s ω = 0.92). The reliability estimates obtained after sequential removal of individual items are presented in Table 5.

Removal of any individual item did not result in an improvement in the reliability of the total score. The internal consistency of the subjective financial situation (Cronbach’s α = 0.90; McDonald’s ω = 0.90) and the disease-related financial strain subscales (Cronbach’s α = 0.87; McDonald’s ω = 0.87) was also high. Table S1 in Supplementary Materials section provides specific data on raw item scores and their discriminant power. Every item reached at least a high level of discriminant power (above 0.50). For the subjective financial situation scale, disease-related strain and the total score, item discriminant power ranged from 0.67 to 0.81, 0.59 to 0.75, and 0.52 to 0.78, respectively. No improvement in internal consistency was observed after removing items, and the high to very high discriminant power suggests good to very good reliability of the COST-FACIT scale. A potential floor effect was observed for a small number of items, indicated by positive skewness and median values close to the minimum. Items 2 and 4 indicated the highest risk of a floor effect and may be less sensitive in differentiating among respondents with lower scores. These items also demonstrated comparatively lower discriminant power, supporting the presence of a potential floor effect. An additional aspect of reliability assessment was the evaluation of relative stability over time. Test–retest reliability was examined using re-administration of the COST-FACIT after a 7–10-day interval (see Table 6).

Strong and very strong correlations between the initial assessment and the re-test confirm the temporal stability (test–retest reliability) of the COST-FACIT instrument. The overall score of the instrument demonstrated a very strong test–retest correlation. Taken together, the high levels of internal consistency and temporal stability support the reliability of the COST-FACIT instrument in the context of endometriosis. Additionally, Wilcoxon test analysis showed that the subjective financial situation subscale in the pre-test (M = 9.93, SD = 4.28) and re-test (M = 9.48, SD = 4.39) showed no significant differences (z = 1.55, *p* = 0.12, r = 0.19). Raw scores obtained for the disease-related financial strain subscale in the pre-test (M = 8.05, SD = 5.34) were higher than in the re-test (M = 7.35, SD = 5.23) (z = 2.33, *p* = 0.02, r = 0.29). Simultaneously, the total score in the pre-test (M = 17.98, SD = 8.84), compared to the re-test (M = 16.83, SD = 8.85), was higher (z = 2.08, *p* = 0.04, r = 0.25). The observed effect sizes were small.

### 3.3. Standardization

The large sample size obtained in the study allowed for the presentation of a normative reference for score interpretation. Due to the non-normal distribution of the overall COST-FACIT endometriosis score (K-S (504) = 0.07, *p* < 0.01), a non-linear transformation of scores was applied (Magnusson, 1981, as cited in Hornowska, 2001) [23,24]. In Table 7, the raw COST-FACIT endometriosis scores are presented along with their corresponding values on the standardized scale.

The values presented in Table 7 should be regarded as a promising, yet preliminary, screening approach to the assessment of financial toxicity. Scores ranging from 1 to 3 sten may be interpreted as indicating high financial toxicity (low financial well-being), whereas scores between 8 and 10 sten reflect low financial toxicity (high financial well-being). Values within the 4–7 sten range may be considered indicative of a moderate level of financial burden.

For example, a patient with a sten score of 2 may experience substantial financial strain related to their condition, while a patient scoring 9 is likely to report minimal financial burden and good financial well-being.

### 3.4. Demographic and Clinical Correlations of COST-FACIT Scores

The overall COST-FACIT illness score was compared with selected demographic and clinical factors related to endometriosis. A summary of the multivariable regression model is presented in Table 8. The model incorporating groups of demographic and clinical variables demonstrated a good fit to the data, explaining less than 30% of the variance in scores.

Among the demographic variables, education level, employment status, and monthly income showed a significant association with the overall COST-FACIT illness score. As illustrated in Figure S1 (see Figure S1 in the Supplementary Materials), women with higher education achieved higher levels of financial well-being in the context of illness. In terms of employment status, the highest COST-FACIT illness scores were observed among women with permanent employment and those on maternity leave, whereas the lowest scores were recorded among unemployed women and those on sick leave. Figure S1 also demonstrates that increasing monthly income was associated with higher levels of financial well-being during illness (COST-FACIT illness). Variables such as age, having children, and marital status were not significant predictors of financial well-being.

Among the clinical variables, time since the onset of first symptoms, distance to the treatment facility, and total treatment-related costs incurred to date were significantly correlated with COST-FACIT illness scores. As shown in Figure S2 (Supplementary Materials), increasing distance from the medical facility and higher total treatment costs were associated with lower levels of financial well-being among women with endometriosis. A similar pattern was observed for time since the onset of symptoms, with longer disease duration corresponding to lower financial well-being. An additional descriptive observation concerned extreme values for time since symptom onset (Max = 35 years) and total treatment costs (Max = 250,000 PLN). In a very small number of cases representing these extremes, relatively higher COST-FACIT illness scores were observed.

## 4. Discussion

The present study provides the first psychometric validation of the COST-FACIT instrument in a population of women with endometriosis. The findings indicate that the Polish adaptation of the scale exhibits good construct validity, strong internal consistency and high temporal stability, supporting its use as a measure of financial toxicity in this non-oncological chronic condition. These results extend the applicability of the COST-FACIT beyond oncology and provide a structured approach to assessing financial burden in endometriosis care from the patient’s perspective.

Construct validity was supported through exploratory and confirmatory factor analyses. All tested models showed an acceptable fit to the data; however, the two-factor solution proposed in the present study demonstrated the best fit. The identified dimensions—subjective financial situation and disease-related financial strain—were strongly correlated, and the one-factor model also showed an acceptable fit. Together, these results support the use of the total COST-FACIT score as the primary outcome measure, consistent with its original conceptualization as a largely unidimensional construct [19]. Similar patterns have been observed in previous validation studies, where strong inter-factor correlations supported the interpretation of the global score [12,19]. These findings are further supported by systematic evidence indicating that financial toxicity is a complex construct, encompassing both objective economic burden and subjective psychosocial distress, and it requires comprehensive psychometric instruments for accurate assessment [10]. At the same time, the good fit of the two-factor model suggests that COST-FACIT may capture distinct but closely related dimensions of financial toxicity [12,19,25].

The emergence of a two-factor structure in both diabetes and endometriosis populations indicates that financial toxicity may inherently comprise multiple interrelated dimensions, such as overall financial situation and disease-specific financial impact. While these subscales should be approached with caution, they may offer additional value in future research aimed at exploring specific domains of financial burden. Contrary to oncology-focused research, in the present study, this conceptual framework is extended to a chronic gynecological condition, further supporting the cross-disease relevance of the instrument [12].

Reliability analyses demonstrated strong internal consistency for the total score, with Cronbach’s alpha and McDonald’s omega coefficients exceeding 0.90. Importantly, removal of any individual item did not improve reliability, indicating a coherent and well-balanced scale structure. High reliability coefficients were also observed for both subscales, consistent with previous COST-FACIT validation studies conducted in oncological and non-oncological populations [12,19]. Furthermore, test–retest analysis confirmed high temporal stability over a 7–10-day interval, supporting the relative stability of the instrument and its suitability for repeated measurement in longitudinal research. These findings further support the robustness of the Polish COST-FACIT in women with endometriosis [14,21].

The large sample size enabled preliminary standardization of COST-FACIT scores among women with endometriosis. Due to the non-normal distribution of the total score, nonlinear transformation was applied, allowing for the presentation of standardized values. The proposed interpretation of sten scores should be regarded as a screening-oriented approach rather than definitive clinical norms. Further research is needed before establishing population-level reference values [10].

The psychometric properties observed in the present study are consistent with findings from previous COST-FACIT validation studies conducted across diverse cultural contexts. Cross-cultural adaptations in oncology populations have consistently exhibited good reliability, construct validity and stable factor structures. Although minor variations in factor structure have been reported across languages, these adaptations uniformly supported the interpretation of a global COST-FACIT score, reflecting strong conceptual equivalence of the instrument. Overall, these findings are consistent with previous validation studies demonstrating good psychometric performance of the COST-FACIT across different cultural contexts [26,27,28,29,30,31].

Originally developed in oncology, the COST-FACIT has increasingly been applied in other chronic conditions characterized by long-term treatment and cumulative economic burden. The present findings further support the conceptual extension of financial toxicity beyond cancer to chronic, non-malignant conditions such as endometriosis. Endometriosis is associated with prolonged diagnostic delays, repeated medical consultations, surgical interventions and productivity loss, all of which may contribute to sustained financial strain. Evidence from other chronic conditions, including diabetes and age-related macular degeneration, further supports the applicability and generalizability of the COST-FACIT across diseases and healthcare systems [12,32].

Beyond psychometric validation, in this study, important insights are provided into demographic and clinical determinants of financial toxicity in women with endometriosis. Our multivariable analysis identified several socioeconomic and clinical factors associated with financial well-being.

Higher education, stable employment and greater monthly income were associated with significantly higher COST-FACIT illness scores, highlighting the central role of socioeconomic resources in buffering the financial impact of chronic disease. These findings are consistent with those reported in prior research, indicating that educational attainment and employment stability are key determinants of health-related quality of life and financial resilience in chronic conditions [4,5,33]. Moreover, in research on endometriosis, substantial direct and indirect costs are consistently reported, including productivity loss, underscoring the vulnerability of economically disadvantaged patients [4,33]. Conversely, unemployment and sick leave were associated with lower COST-FACIT illness scores, indicating greater financial toxicity and reflecting the bidirectional relationship between disease burden and labor market participation.

From a clinical perspective, longer time since symptom onset, greater distance to treating healthcare facilities and higher cumulative treatment costs were associated with reduced COST-FACIT illness scores, indicating that prolonged diagnostic pathways and structural barriers to care may exacerbate financial vulnerability. These results align with existing evidence, suggesting that delayed diagnosis and fragmented access to specialist services contribute to both economic strain and poorer patient-reported outcomes in endometriosis [4,6,7,8,9,33].

Although the regression model demonstrated a good overall fit, it explained less than 30% of the variance in financial well-being, indicating that substantial unexplained heterogeneity remains. This likely reflects the multifactorial nature of financial toxicity in endometriosis, including psychosocial factors, social support, coping strategies and healthcare system characteristics not captured in the present study.

Extreme values were observed for both disease duration and cumulative healthcare expenditures. While these cases represented only a small subset of participants, they highlight variability in individual disease trajectories and adaptive responses, warranting further investigation in longitudinal studies. These findings emphasize that financial toxicity in endometriosis is shaped by a complex interplay of socioeconomic position and disease-related factors. Similar patterns have been reported for chronic disease populations, in which lower socioeconomic status, limited access to care and prolonged disease burden are consistently associated with worse patient-reported outcomes and increased financial strain [10,12,19]. This supports the need for integrated clinical and social care approaches addressing both the medical and economic dimensions of the condition. In addition, these results have important policy implications, highlighting the need for improved healthcare financing models.

### Limitations

Despite efforts to address potential sources of bias, several limitations of this study should be acknowledged. First of all, the sample was recruited online and on a voluntary basis, resulting in a self-selected population that may not be fully representative of the broader population of women with endometriosis. Participants with higher education were substantially overrepresented (71.6%), which may have influenced both health literacy and financial coping strategies, potentially inflating observed levels of financial well-being. This limits the generalizability of the findings, particularly to populations with lower educational attainment and different socioeconomic conditions. This pattern is common in online recruitment studies, which tend to attract individuals with higher digital literacy and greater engagement in health-related topics, and may also partly reflect broader demographic trends observed in Poland. Secondly, all clinical data were self-reported, and although eligibility criteria were aligned with international guidelines and responses were reviewed for consistency, clinical verification was not possible. Furthermore, the inclusion of both confirmed and self-reported (suspected) cases of endometriosis may have introduced additional heterogeneity and potential misclassification bias, as not all cases were surgically or clinically verified. Consequently, residual misclassification and reporting bias cannot be entirely excluded. This may have influenced the observed associations and potentially attenuated the strength of the relationships identified in the analysis. In this study, a distinction was not made between confirmed and suspected diagnoses of endometriosis, which precluded subgroup analyses and may have introduced additional heterogeneity. Thirdly, the cross-sectional design precludes causal inference. While significant associations were noted between financial well-being and selected demographic and clinical variables, temporal relationships cannot be established. Fourthly, although multivariable regression was used to account for key demographic and clinical factors, the final model explained less than 30% of the variance in COST-FACIT scores, indicating that a substantial proportion of variability remains unexplained. Unmeasured factors, such as psychological resilience, social support or differences in healthcare access, may have contributed to financial well-being, but were not captured in the present analysis. Additionally, extreme values were observed for both time since symptom onset and total healthcare expenditures. Although these cases represented only a small share of the sample, they may have influenced overall estimates and reflected heterogeneity in disease trajectories that could not be fully explored within the current study framework. Although exploratory and confirmatory factor analyses were conducted in two independent randomly selected subsamples, external validation in an independent clinical sample remains necessary. Therefore, the proposed factor structure should be interpreted as internally validated but still requiring confirmation in future studies. Taken together, these limitations suggest that the findings should be interpreted with caution and primarily generalized to populations similar to the present sample. Future studies using clinically verified diagnoses, longitudinal designs and more socioeconomically diverse samples are warranted.

## 5. Conclusions

In conclusion, the Polish version of the COST-FACIT demonstrates good psychometric properties in women with endometriosis and may serve as a useful instrument for assessing financial toxicity in this population. While the total score is recommended as the primary outcome measure, the identified subdimensions may provide additional insights in future research. The availability of a validated tool for assessing financial toxicity in endometriosis is a significant step towards better understanding the economic burden of this condition. Its use may facilitate the identification of patients at risk of financial distress, support more individualized clinical decision-making and enable the integration of financial toxicity assessment into routine care. At a system level, these findings may inform healthcare planning, reimbursement strategies and the development of targeted patient support programs aimed at reducing the long-term economic burden of endometriosis.

## Figures and Tables

**Table 1:** Characteristics of the study group.

Variables			Main Group N = 504		Re-Test Group N = 82	
			N	%	N	%
Sociodemographic	Age	18–25 years	77	15.28	21	25.61
		26–35 years	228	45.24	33	40.24
		36–45 years	166	32.94	24	29.27
		Above 45 years	33	6.55	4	4.88
	Education level	Primary	16	3.17	1	1.22
		Secondary	127	25.20	21	25.61
		Higher	361	71.63	60	73.17
	Marital status	Single	103	20.44	22	26.83
		Informal partnership	119	23.61	22	26.83
		Married	260	51.59	34	41.46
		Divorced/widowed	22	4.37	4	4.88
	Children	No	329	65.28	60	73.17
		Yes	175	34.72	22	26.83
	Employment status	Unemployed	50	9.92	7	8.54
		Temporary	30	5.95	6	7.32
		Maternity leave	21	4.17	3	3.66
		Sick leave	25	4.96	3	3.66
		Permanent	378	75.00	63	76.83
	Income [PLN]	Below 3000	80	15.87	11	13.41
		3000–5000	141	27.98	22	26.83
		5001–8000	157	31.15	29	35.37
		Above 8001	126	25.00	20	24.39
Clinical Factors	Age at diagnosis ^a^	Below 26 years	150	29.76	32	39.02
		26–35 years	250	49.60	38	46.34
		36–45 years	95	18.85	11	13.41
		Above 45 years	6	1.19	1	1.22
	Time since first symptoms [years]	Below 5	158	31.35	22	26.83
		5–10	150	29.76	28	34.15
		11–14	73	14.48	12	14.63
		Above 15	123	24.40	20	24.39
	Distance to treating healthcare facility [km] ^b,d^	Below 7	139	27.58	22	26.83
		7–20	124	24.60	16	19.51
		21–70	116	23.02	21	25.61
		Above 71	122	22.21	21	25.61
	Infertility treatment	No	369	73.21	61	74.39
		Yes	135	26.79	21	25.61
	Healthcare costs [PLN] ^c^	Below 2000	131	25.99	24	29.27
		2000–6000	130	25.79	20	24.39
		6001–20,000	133	26.39	15	18.29
		Above 20,000	109	21.63	23	28.05

Note. Missing values (0.59% n = 3) ^a,b^, (0.20% n = 1) ^c^ in main group, (2.22% n = 2) ^d^ in re-test group. N—number of observations.

**Table 2:** Factor loadings of COST-FACIT illness [endometriosis] scale.

COST-FACIT Illness [Endometriosis]	Factor 1	Factor 2
Item 1	**0.73**	−0.09
Item 2	−0.05	**0.67**
Item 3	0.11	**0.72**
Item 4	−0.19	**0.75**
Item 5	0.26	**0.54**
Item 6	**0.79**	0.07
Item 7	**0.79**	−0.05
Item 8	**0.52**	0.36
Item 9	0.32	**0.41**
Item 10	0.25	**0.59**
Item 11	**0.81**	0.05

**Table 3:** Fit index coefficients for COST-FACIT illness [endometriosis] scale.

Tested Model	χ^2^	df	*p*	χ^2^/df	RMSEA	95% CI		CFI	TLI
						LL	UL		
One-factor	139.39	44	<0.01	3.17	0.07	0.05	0.08	0.93	0.91
Two-factor ^a^	167.58	43	<0.01	3.90	0.05	0.05	0.06	0.97	0.96
Two-factor ^b^	131.17	43	<0.01	3.05	0.06	0.05	0.08	0.93	0.91

^a^—based on previous Exploratory Factor Analysis, ^b^—based on the COST-FACIT diabetes model [12].

**Table 4:** Correlation analysis of Polish COST-FACIT illness [endometriosis] scale with Financial Well-Being.

COST-FACIT Illness [Endometriosis]	Financial Well-Being	
	r_s_	*p*
Subjective financial situation	0.84	<0.01
Disease-related financial strain	0.64	<0.01
Total score	0.79	<0.01

Note. Number of follow-up observations, N = 504. r_s_—Spearman’s coefficient, *p*—significance.

**Table 5:** Internal consistency of the Polish COST-FACIT illness [endometriosis] scale while removing items.

Item	Subjective Financial Situation		Disease-Related Financial Strain		Total Score	
	α_C_	ω_M_	α_C_	ω_M_	α_C_	ω_M_
1	0.89	0.89			0.91	0.92
2			0.85	0.86	0.92	0.92
3			0.83	0.83	0.91	0.91
4			0.86	0.86	0.92	0.92
5			0.84	0.84	0.91	0.91
6	0.86	0.86			0.91	0.91
7	0.88	0.88			0.91	0.91
8	0.88	0.88			0.91	0.91
9			0.85	0.85	0.91	0.91
10			0.83	0.83	0.91	0.91
11	0.86	0.86			0.91	0.91

Note. Number of observations, N = 504. α_C_—Cronbach’s alpha, ω_M_—McDonald’s omega.

**Table 6:** Correlation analysis of Polish COST-FACIT illness [endometriosis] scale with 7–10-day follow-up.

COST-FACIT Illness [Endometriosis]	Follow-Up	
	r_s_	*p*
Subjective financial situation	0.83	<0.01
Disease-related financial strain	0.85	<0.01
Total score	0.85	<0.01

Note. Number of follow-up observations, N = 82. r_s_—Spearman’s coefficient, *p*—significance.

**Table 7:** Standard values of Polish COST-FACIT illness [endometriosis] total score.

Raw Score [Min–Max]	Standard Sten Scale
0	1
1–2	2
3–6	3
7–10	4
11–15	5
16–20	6
21–26	7
27–31	8
32–36	9
37–44	10

**Table 8:** General linear model predicting Polish COST-FACIT illness [endometriosis] results.

Antecedent			COST-FACIT Illness [Endometriosis]							
			B	SE	t	β	CI 95%		*p*	VIF
							LL	UL		
Constant			10.48	2.28	4.60				<0.01	
Sociodemographic	Age		0.01	0.08	0.17	0.01	−0.12	0.14	0.87	1.78
	Education level	No higher → Higher	2.57	0.90	2.86	**0.27**	**0.08**	**0.45**	**<0.01**	**1.12**
	Marital status	Single → Informal partnership	−0.77	1.11	−0.70	−0.08	−0.31	0.45	0.49	1.12
		Single → Married	0.61	1.16	0.52	0.06	−0.18	0.30	0.60	
		Single → Divorced/widowed	−0.65	2.03	−0.32	−0.07	−0.49	0.35	0.75	
	Children	No → Yes	1.15	1.09	1.05	0.12	−0.10	0.35	0.29	1.44
	Employment status	Permanent → Unemployed	−5.04	1.38	−3.64	**−0.53**	**−0.81**	**−0.24**	**<0.01**	**1.07**
		Permanent → Temporary	−1.33	1.63	−0.82	-0.14	−0.47	0.20	0.42	
		Permanent → Maternity leave	1.35	1.95	0.69	0.14	−0.26	0.54	0.49	
		Permanent → Sick leave	−7.25	1.70	−4.26	**−0.76**	**−1.11**	**−0.41**	**<0.01**	**1.08**
	Income [PLN]	Below 3000 → 3001−5000	3.59	1.25	2.86	**0.38**	**0.12**	**0.63**	**<0.01**	
		Below 3000 → 5001−8000	5.38	1.30	4.13	**0.56**	**0.30**	**0.83**	**<0.01**	
		Below 3000 → Above 8001	9.99	1.39	7.19	**1.05**	**0.76**	**1.33**	**<0.01**	
Clinical Factors	Age at diagnosis		0.07	0.09	0.80	0.05	-0.07	0.17	0.42	1.65
	Time since first symptoms [years]		−0.21	0.06	−3.60	**−0.15**	**−0.22**	**−0.07**	**<0.01**	**1.06**
	Distance to treating healthcare facility [km]		−0.01	0.00	−2.07	**−0.08**	**−0.16**	**0.00**	**0.04**	**1.06**
	Infertility treatment	No → Yes	−1.26	0.95	−1.33	−0.13	-0.33	0.06	0.19	1.16
	Healthcare costs [PLN]		0.00	0.00	−2.76	**−0.12**	**−0.20**	**−0.03**	**<0.01**	**1.10**
Model fit			R^2^_s_ = 0.29, F_(18, 478)_ = 12.04, *p* < 0.01							

B—non-standardized coefficient, SE—standard error, t—Student’s statistic, β—standardized coefficient, CI—confidence interval, LL—lower limit, UL—upper limit, *p*—significance, F—ANOVA model fit, R^2^_s_—adjusted determination coefficient, VIF—variance inflation index.

## Data Availability

The original contributions presented in the study are included in the article/Supplementary Materials; further inquiries can be directed to the corresponding author.

## Supplementary Materials

The following supporting information can be downloaded at: https://www.mdpi.com/article/10.3390/healthcare14111449/s1, Figure S1: Mean and standard error results of Polish COST-FACIT illness [endometriosis] according to selected sociodemographic factors; Figure S2: Mean and standard error results of Polish COST-FACIT illness [endometriosis] according to selected clinical factors. Table S1: Item-level descriptive statistics and discrimination indices for the COST-FACIT scale.

## Abbreviations

The following abbreviations are used in this manuscript:

NFZ	Narodowy Fundusz Zdrowia [National Health Fund]
FACIT-COST	Functional Assessment of Chronic Illness Therapy—Comprehensive Score for Financial Toxicity
MRI	Magnetic Resonance Imaging
ABM	Agencja Badań Medycznych [Polish Medical Research Agency]
GDPR	General Data Protection
CFPB	Consumer Financial Protection Bureau
FWBS	Financial Well-Being Scale
EFA	Exploratory Factor Analysis
CFA	Confirmatory Factor Analysis
GLM	Generalized Linear Model
