# Supplementary material for: Financial Toxicity in Women with Endometriosis: Psychometric Validation of the Polish COST-FACIT with Analysis of Demographic and Clinical Factors
Source: Healthcare (Basel). 2026 May 24;14(11):1449. doi: 10.3390/healthcare14111449 (PMC13257366; doi:10.3390/healthcare14111449)
Supplement: Supplementary file 1 [file healthcare-14-01449-s001.zip › healthcare-4254868-supplementary.pdf]

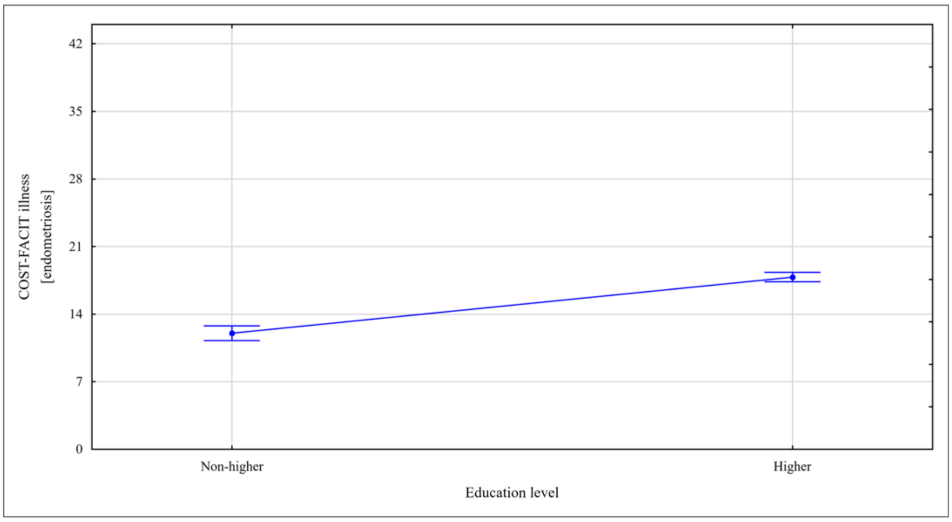

A. Considering education level.

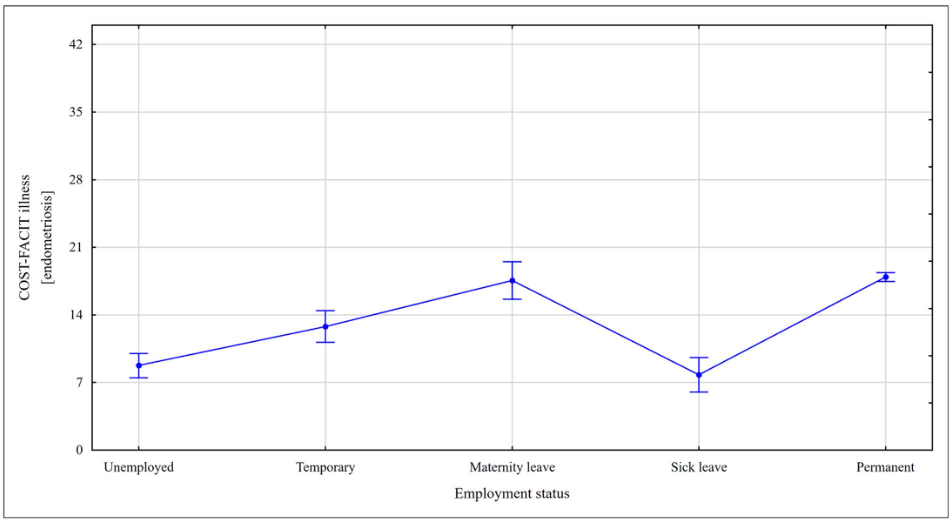

B. Considering employment status.

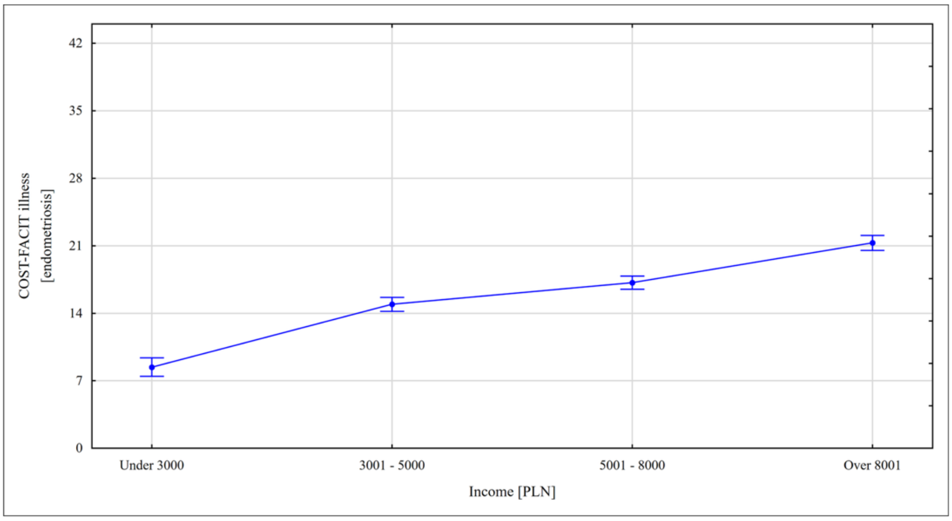

C. Considering income.

Figure S1. Mean and standard errors results of Polish COST-FACIT illness [endometriosis] according to selected sociodemographic factors: (A) education level, (B) employment status, and (C) income.

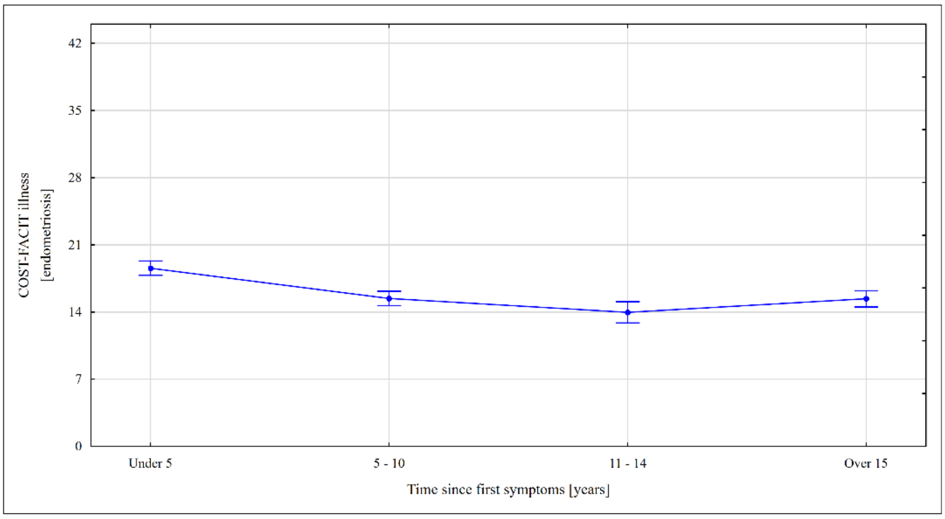

A. Considering time since first symptoms.

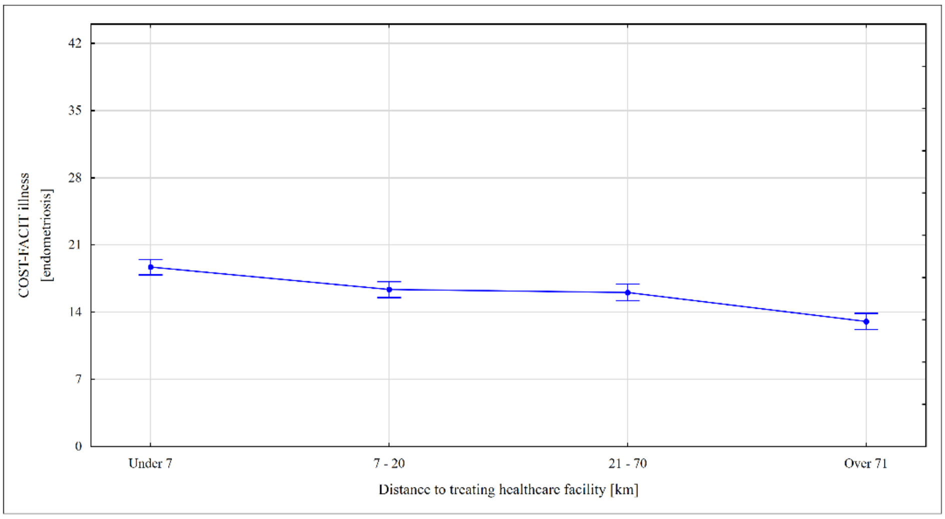

B. Considering distance to treating healthcare facility.

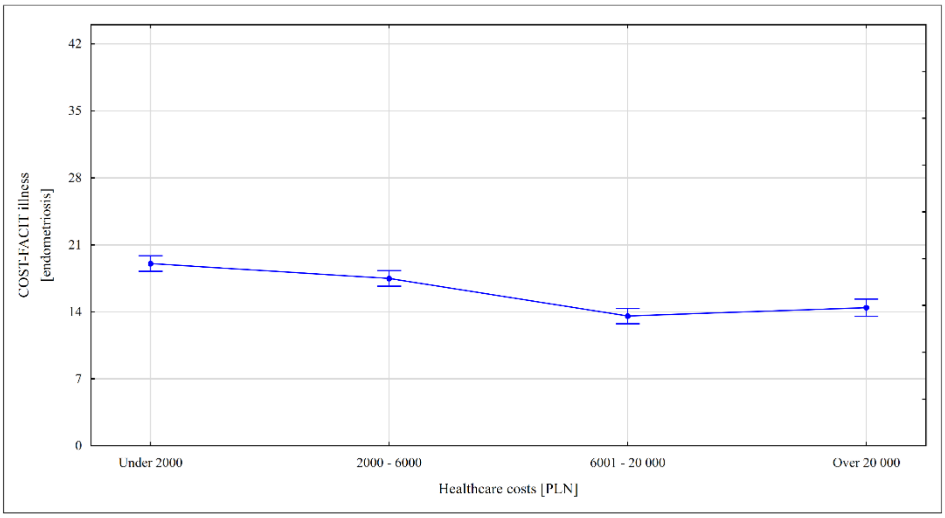

C. Considering healthcare costs.

Figure S2. Mean and standard errors results of Polish COST-FACIT illness [endometriosis] according to selected clinical factors: (A) time since first symptoms, (B) distance to treating healthcare facility, and (C) healthcare costs.

Table S1. Item-level descriptive statistics and discrimination indices for COST-FACIT scale

COST-FACIT illness [endometriosis]	Descriptive statistics							Discriminant power		
	Min	*Max*	*M*	*Me*	*SD*	*SKE*	*K*	Subjective financial situation	Disease-related financial strain	Total score
Item 1	0.00	4.00	1.34	1.00	1.12	0.28	-0.88	0.67		0.62
Item 2	0.00	4.00	0.94	1.00	1.05	1.02	0.51		0.59	0.54
Item 3	0.00	4.00	1.09	1.00	1.16	0.75	-0.41		0.74	0.74
Item 4	0.00	4.00	0.91	1.00	1.05	1.07	0.61		0.59	0.52
Item 5	0.00	4.00	1.35	1.00	1.34	0.57	-0.89		0.68	0.68
Item 6	0.00	4.00	1.70	2.00	1.16	0.04	-0.86	0.81		0.75
Item 7	0.00	4.00	2.53	3.00	1.12	-0.60	-0.16	0.73		0.66
Item 8	0.00	4.00	1.47	2.00	1.18	0.19	-0.99	0.70		0.78
Item 9	0.00	4.00	1.69	2.00	1.33	0.18	-1.10		0.63	0.68
Item 10	0.00	4.00	1.27	1.00	1.23	0.60	-0.66		0.75	0.77
Item 11	0.00	4.00	1.90	2.00	1.10	-0.13	-0.63	0.80		0.74
